# Flotillin proteins recruit sphingosine to membranes and maintain cellular sphingosine-1-phosphate levels

**DOI:** 10.1371/journal.pone.0197401

**Published:** 2018-05-22

**Authors:** Kirsi Riento, Qifeng Zhang, Jonathan Clark, Farida Begum, Elaine Stephens, Michael J. Wakelam, Benjamin J. Nichols

**Affiliations:** 1 Medical Research Council Laboratory of Molecular Biology, Cambridge, United Kingdom; 2 The Babraham Institute, Babraham Research Campus, Cambridge, United Kingdom; Universite Paris Diderot-Paris7 - Batiment des Grands Moulins, FRANCE

## Abstract

Sphingosine-1-phosphate (S1P) is an important lipid signalling molecule. S1P is produced via intracellular phosphorylation of sphingosine (Sph). As a lipid with a single fatty alkyl chain, Sph may diffuse rapidly between cellular membranes and through the aqueous phase. Here, we show that the absence of microdomains generated by multimeric assemblies of flotillin proteins results in reduced S1P levels. Cellular phenotypes of flotillin knockout mice, including changes in histone acetylation and expression of Isg15, are recapitulated when S1P synthesis is perturbed. Flotillins bind to Sph *in vitro* and increase recruitment of Sph to membranes in cells. Ectopic re-localisation of flotillins within the cell causes concomitant redistribution of Sph. The data suggest that flotillins may directly or indirectly regulate cellular sphingolipid distribution and signalling.

## Introduction

Sphingosine-1-phosphate (S1P) is a potent signalling molecule, and regulates processes including inflammation, metabolism, vascular permeability, and memory [1–6]. S1P is produced from sphingosine by two related sphingosine kinases (SphKs) [4]. Complete loss of S1P biosynthesis in mice lacking both SphKs is lethal due to neural and vascular developmental defects [7].

Sub-cellular compartmentalisation is likely to play important roles in the biosynthesis of S1P and hence downstream signalling. Both SphKs use membrane-associated Sph as substrate [8], but have different sub-cellular distributions. SphK1 is recruited to the plasma membrane while SphK2 has been observed on different intracellular membranes, and inside the nucleus [2, 4, 6].

Given the above, the biophysical properties of both Sph and S1P are intriguing. Both lipids have single alkyl chains, and so are partially soluble in the aqueous phase [9–11]. Sph, which has a less charged head-group than S1P, can flip within bilayers and hence has the potential to distribute throughout all the membranes of the cell.

Flotillin 1 and flotillin 2 co-assemble to form multimeric protein complexes, or microdomains, on the cytosolic face of cell membranes. These microdomains are present in the plasma membrane, and on the membranes of late endosomes and lysosomes [12–14]. Flotillins are multiply acylated and have patches of hydrophobic amino acid residues that are likely to be embedded within the bilayer. Both flotillin proteins are required for multimerisation [13]. Additional evidence for functional co-dependency between flotillin proteins comes from studies on single and double knockout mice [15]. Flotillins have been implicated in various cellular processes, but relevant cellular and molecular mechanisms have not been determined [14, 16–20].

This study was initiated by lipidodomic characterisation of cultured cells and tissues from flotillin 1 knockout (*Flot1*^-/-^) and in flotillin 1 and flotillin 2 double knockout (*Flot1*^-/-^, *Flot2*^-/-^) mice. The most prominent change in the knockouts compared to congenic controls was reduced S1P levels. Some of the cellular and physiological phenotypes of flotillin knockouts can be recapitulated by perturbing S1P signalling. Flotillins bind to sphingosine and control its sub-cellular distribution. Our data suggest a potential link between flotillins and sphingolipid-dependent signalling processes.

## Results

### Lipidomic profile of flotillin knockout cells

**Fig 1:**
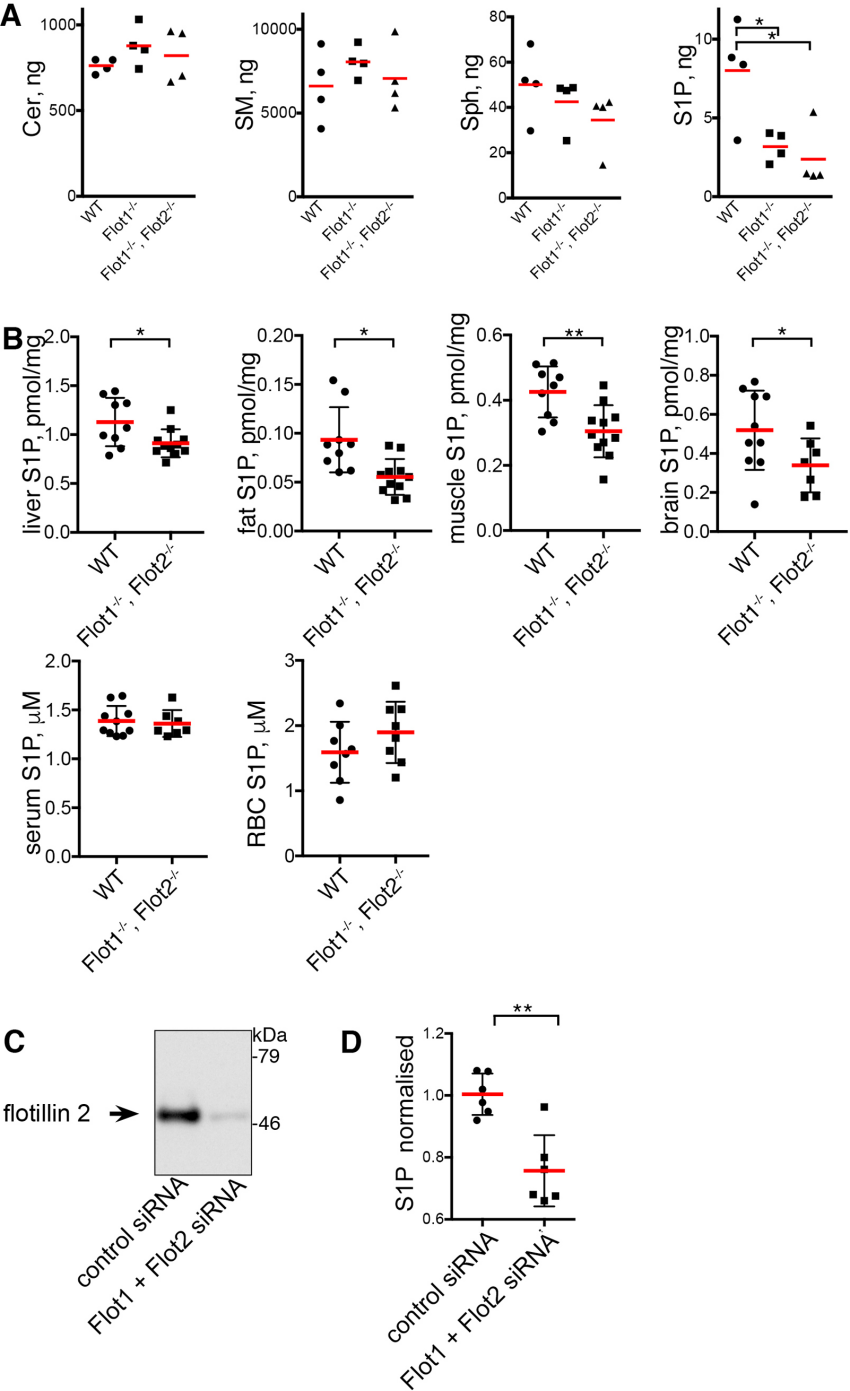
Loss of flotillins causes a reduction in cellular and tissue sphingosine-1-phosphate. **A.** Quantitative lipid mass spectrometry using calibration with defined standards, of WT and flotillin knockout MEFs. 1x10^6^ cells were analysed in each sample.; Cer, Ceramide; SM, sphingomyelin; Sph, sphingosine; S1P, sphingosine-1-phosphate. Data from 4 biological replicates, each data point being derived from a separate experiment. Bars are mean. Statistical test is one-way ANOVA with Dunnett’s multiple comparisons test. **B.** S1P levels in mouse tissues and serum as shown, determined by quantitative lipid mass spectrometry. Bars are means and SD, each point represents a single sample from a different animal. Student’s t-test. **C**. Western blot to show reduction in flotillin 2 expression in cells treated with siRNAs against flotillin 1 and flotillin 2. Full scans of the blot are included in S3 Data File. **D**. siRNAs against flotillin 1 and flotillin 2 cause a reduction in S1P levels in HeLa cells as determined by quantitative lipid mass spectrometry. Data are normalised so that the mean of the control samples = 1, the data shown are from two separate experiments, in each experiment 3 biological replicates were analysed. Bars are mean and SD. Student’s t-test.

We compared levels of multiple cellular lipids in Folch extracts of primary mouse embryonic fibroblasts (MEFs) from congenic wild type (WT) and flotillin knockout mice. Mass spectrometry data revealed a significant reduction in sphingosine-1-phosphate (S1P) levels in flotillin 1 knockout (*Flot1*^-/-^) and in flotillin 1 and flotillin 2 double knockout (*Flot1*^-/-^, *Flot2*^-/-^) cells (Fig 1A). There was no difference between *Flot1*^*-/-*^ and *Flot1*^*-/-*^, *Flot2*^*-/-*^ cells in this or subsequent experiments. Levels of all 18 other lipids analysed were not detectably altered (Fig 1A, S1 Fig, S1 Data File and S2 Data File), and both the length and saturation of acyl chains in the abundant sphingolipids ceramide and sphingomyelin were completely indistinguishable in WT and *Flot1*^*-/-*^, *Flot2*^*-/-*^ cells (S2 Fig).

We next determined S1P levels in mouse tissues, including white adipose tissue, muscle, liver and brain, using n-butanol extraction. Significantly reduced levels of S1P were found in all *Flot1*^-/-^, *Flot2*^-/-^ tissues analysed (Fig 1B). Sph levels in these tissues were not detectably altered (S3 Fig). Serum and red blood cells (RBCs) have a high concentration of S1P [6, 9]. The serum and RBC S1P levels we assayed were comparable with those observed previously [6, 21, 22], and were not significantly altered in *Flot1*^-/-^, *Flot2*^-/-^ mice (Fig 1B). As blood was still present in the tissue samples, and there is more S1P in blood than in tissue parenchyma, it is possible that S1P levels in tissue parenchyma are more substantially reduced in *Flot1*^-/-^, *Flot2*^-/-^ mice than is reflected in our measurements [6].

As an additional test of whether more acute perturbation of flotillin microdomains reduces cellular S1P, we carried out siRNA experiments in HeLa cells. Knockdown of flotillin 1 and 2 expression [23] caused a significant reduction in S1P levels in n-butanol extracts (Fig 1C and 1D). Loss of flotillins is therefore associated with reduced cellular S1P levels in multiple cell culture systems and tissues.

### Phenotypes of flotillin knockouts reflect altered S1P levels

**Fig 2:**
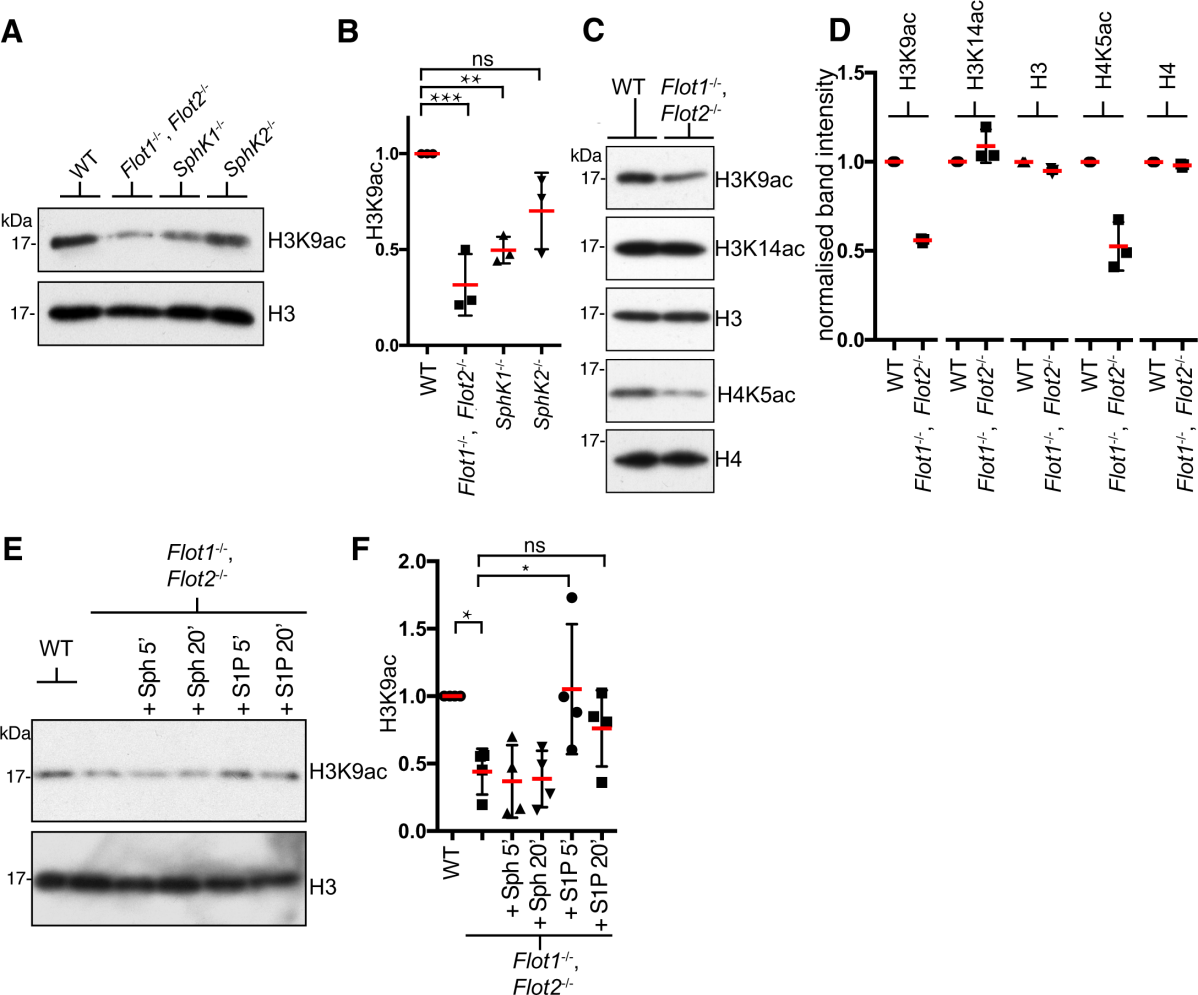
Deletion of flotillin genes replicates a known cellular phenotype of reduced S1P. **A** Histone H3K9 acetylation in WT, *Flot1*^-/-^, *Flot2*^-/-^, *SphK1*^*-/-*^ and *SphK2*^*-/-*^ primary MEFs was analysed by Western blotting. **B**. Blots from 3 separate experiments quantifying H3K9 acetylation as A were quantified by densitometric scanning, and values normalised so that intensity in the WT sample = 1. Bars are SD. The same normalisation procedure is carried out in all quantitative graphs in this Fig. Significance is assessed using one-way ANOVA and Dunnett’s multiple comparisons test. **C** Histone expression and acetylation in WT and *Flot1*^-/-^, *Flot2* primary MEFs was analysed by Western blotting using the antibodies shown. **D**. Blots from 3 separate experiments quantifying histone acetylation as C were quantified by densitometric scanning. Bars are SD. **E**. Isolated nuclei were treated with 20 **μ**M Sph or S1P as shown. Samples were then analysed by Western blotting to detect total histone H3 and histone H3K9 acetylation. Blots representative of 4 experiments are shown. **F.** Quantification of H3K9 acetylation as E, Bars SD. Significance is assessed using one-way ANOVA and Dunnett’s multiple comparisons test to compare the untreated *Flot1*^-/-^, *Flot2* sample with all other samples.

We sought to test whether flotillin knockout cells display alterations in processes previously shown to be regulated by cellular S1P. S1P has been reported to bind to and inhibit the activity of histone deacetylases HDAC1 and HDAC2, and perturbation of SphKs causes a specific set of alterations in histone acetylation [1, 24]. We confirmed that reduced S1P biosynthesis results in reduced histone 3 K9 acetylation in MEFs by Western blotting of MEF cell extracts from *SphK1*^*-/-*^ and *SphK2*^*-/-*^ mice, and the same reduction was observed in *Flot1*^-/-^, *Flot2*^-/-^ cells (Fig 2A and 2B). Next we assayed additional changes in histone acetylation reported to occur when S1P signalling is perturbed [1, 24]. *Flot1*^-/-^, *Flot2*^-/-^ cells showed the same pattern of changes in histone acetylation as reported in cells treated with SphK siRNAs, with reduced histone 3 K9 and histone 4 K5 acetylation but no change in histone 3 K14 acetylation (Fig 2C and 2D)[1, 24]. Flotillins are, therefore, likely to affect histone acetylation through alterations in S1P levels, and reduced S1P levels were indeed detected in isolated nuclei from *Flot1*^-/-^, *Flot2*^-/-^ cells (levels in n-butanol extracts of *Flot1*^-/-^, *Flot2*^-/-^ nuclei were on average 69% of WT levels, N = 3) These observations predict that the deficit in histone acetylation in *Flot1*^-/-^, *Flot2*^-/-^ cells should be rescued by addition of S1P. Addition of S1P to isolated nuclei from these cells revealed that this is indeed the case (Fig 2E and 2F)[24].

**Fig 3:**
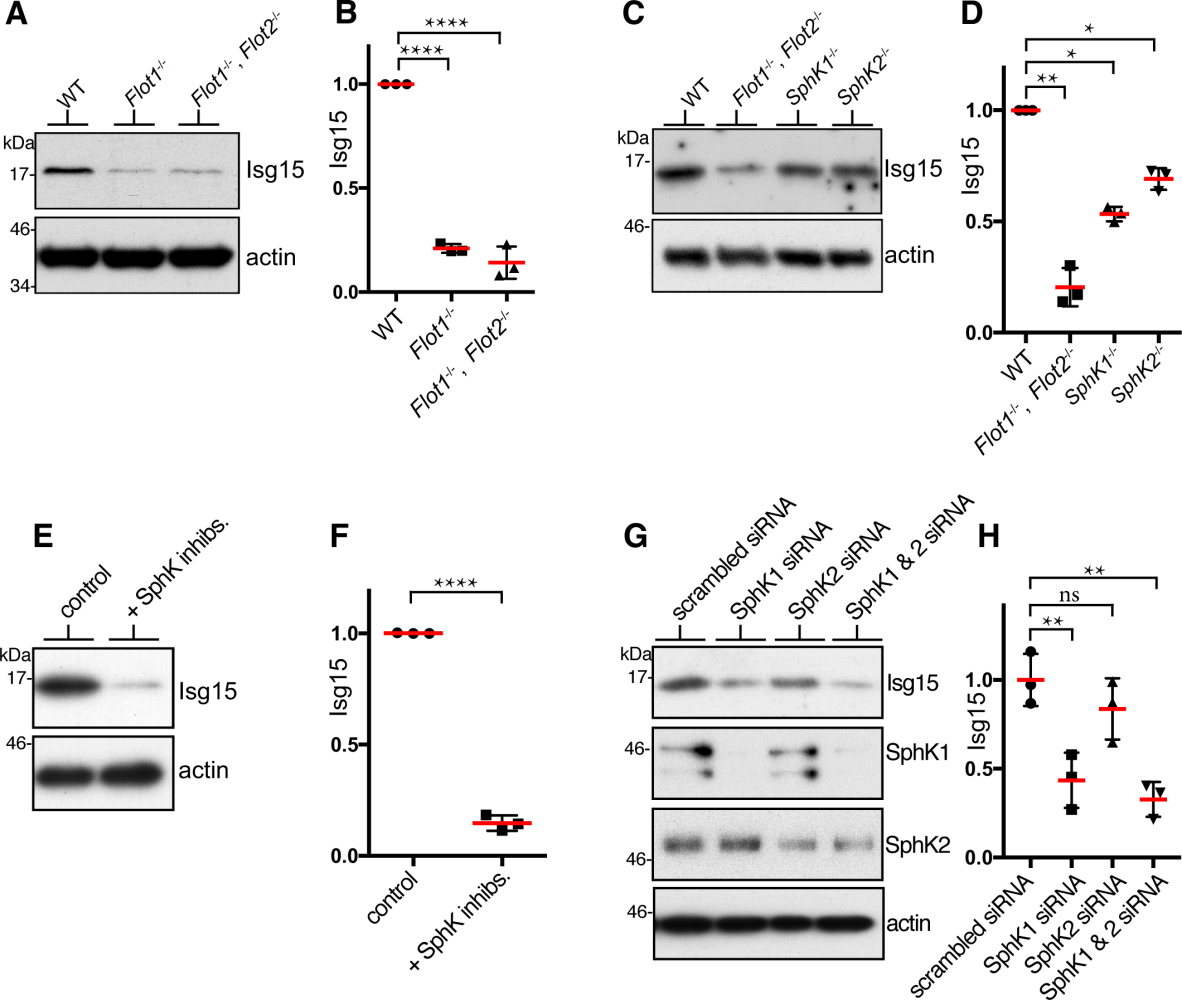
Isg15 levels are reduced in *flotillin* knockouts, and after perturbation of SphK activity. **A.** Isg15 protein expression in WT and *Flot1*^-/-^, *Flot2*^-/-^ primary MEFs was analysed by Western blotting using antibodies against the indicated proteins. **B**. Densitometric quantification of Isg15 expression as A, Bars SD. One-way ANOVA and Dunnett’s multiple comparisons test. **C.** Isg15 protein expression in WT, *Flot1*^-/-^ *Flot2*^-/-^, *Sphk1*^*-/*-^, and *Sphk2*^*-/-*^ primary MEFs was analysed by Western blotting using antibodies against the indicated proteins. **D**. Quantification of Isg15 expression as C, Bars SD. One-way ANOVA and Dunnett’s multiple comparisons test. **E**. Isg15 expression levels in WT MEFs cells treated with SphK1 inhibitor PF543 (10 **μ**M) and with SphK2 inhibitor ABC294640 (50 **μ**M) for 24h as shown. **F**. Quantification of Isg15 expression as E, Bars SD. Student’s t-test. **G**. Isg15 expression in HeLa cells transfected with siRNAs to reduce expression of SphK1 and SphK2. **H**. Quantification of Isg15 expression as G, Bars SD. One-way ANOVA and Dunnett’s multiple comparisons test. Full scans of all blots are shown in S3 Data File.

To gain new insight into cellular functions of flotillins, we analysed changes in protein expression in *Flot1*^-/-^ MEFs compared to WT cells using stable isotope labelling of amino acids in culture (SILAC) [25]. Loss of flotillin 1 resulted in specific changes to protein expression levels (Table 1). Flotillin 2 expression was diminished in *Flot1*^-/-^ cells. Two further proteins had a reduction in expression comparable to that observed for flotillin 2, interferon-stimulated gene product Isg15 and Bst-2/tetherin/CD317. Decreased Isg15 expression was confirmed in *Flot1*^-/-^ and *Flot1*^-/-^, *Flot2*^-/-^ MEFs by Western blotting (Fig 3A and 3B). Quantitative real-time PCR revealed that for both Isg15 and Bst-2 reduced expression correlated with reduced mRNA levels, and hence may be explained by transcriptional changes (S4 Fig).

**Table 1:** SILAC mass spectrometry to identify changes in protein expression levels in *Flot1*^-/-^ cells.

Protein_ID	Protein_Name	Gene_ Name	H/L Ratio A	H/L Ratio B	H/L Ratio C
Q64339	Interferon-stimulated protein 15	Isg15	0.19931	0.21405	0.74012
Q8R2Q8	Bone marrow stromal antigen 2	Bst2	0.22419	0.25447	1.07750
Q60634	Flotillin-2	Flot2	0.30234	0.29526	0.39256
Q9JKZ2	Solute carrier family 5 member 3	Slc5a3	0.44305	0.82481	0.78794
E9Q55	Ring finger protein 213	Rnf213	0.46709	0.36040	1.08810
Q8R1S9	Solute carrier family 38 member 4	Slc38a4	0.65785	0.98883	0.93633
P18572	Basigin	Bsg	0.64210	0.53755	1.02380
Q8C0I1	Alkylglycerone-phosphate synthase	Agps	0.69536	0.99206	0.94860
Q80YX1	Tenascin	Tnc	0.70862	0.95785	0.44446
Q91VH2	Sorting nexin-9	Snx9	0.77664	0.88207	0.81726
P15379	CD44	Cd44	0.77357	0.80821	0.51437
O08573	Galectin-9	Lgals9	0.79942	0.86333	0.96927
P07091	Protein S100-A4	S100a4	0.69401	1.00891	0.66000
P46061	Ran GTPase-activating protein 1	Rangap1	0.79346	0.74212	0.83037
Q61263	Sterol O-acyltransferase 1	Soat1	0.79239	0.93145	0.73752
P09055	Integrin beta-1	Itgb1	0.82048	0.90017	0.70445
P17426	AP-2 complex subunit alpha-1	Ap2a1	0.86333	0.92653	1.07550
Q61171	Peroxiredoxin-2	Prdx2	1.14050	1.37574	1.30410
Q6P1B1	X-prolyl aminopeptidase 1	Xpnpep1	1.15058	1.26493	1.10140
O09061	Proteasome subunit beta type-1	Psmb1	1.10703	1.15611	1.12250
P39876	Metalloproteinase inhibitor 3	Timp3	1.35333	2.20080	1.35280
Q8K4Z3	Apolipoprotein A-I-binding protein	Apoa1bp	1.31751	1.33002	1.18000
Q64429	Cytochrome P450 1B1	Cyp1b1	1.47050	1.18152	3.01490
Q8K173	Collagen alpha-1(III) chain	Col3a1	1.37982	1.26236	1.29440
Q9QXB9	Developmentally-regulated GTP-binding protein 2	Drg2	2.11586	1.78699	1.45530
Q91VE0	Solute carrier family 27 member 4	Slc27a4	2.19414	1.56362	1.14110

Protein mass spectrometry of WT and *Flot1*^-/-^ MEFs labelled with heavy (H) or light (L) amino acids. The proteins were identified and qualified with a minimum of two unique peptides. Quantity of a protein in *Flot1*^-/-^ cells compared to WT cells is shown as H/L ratio. Approximately 1,500 proteins were identified in each of the three (A, B, C) experiments, and of the same 603 proteins identified in all three experiments, only ones that had either reduced or increased ratio are shown.

Isg15 is a ubiquitin-like protein with roles in anti-viral immunity [26]. Identification of a protein which is clearly down-regulated in flotillin knockout cells allowed us to ask whether this cellular phenotype can be caused by reduced S1P levels. We analysed expression of Isg15 in MEFs from control, *SphK1*^*-/-*^ mice and *SphK2*^*-/-*^ mice [7, 27]. Isg15 levels were lower in the knockout MEFs (Fig 3C and 3D). Deletion of both *SphK* genes simultaneously is lethal [7], so we used combined SphK1 and SphK2 inhibitors (PF-543 and ABC294640) to perturb both kinases simultaneously [28]. The inhibitors caused a drop in expression of Isg15, and so replicated both the effect of loss of flotillins and loss of SphKs (Fig 3E and 3F). Use of the inhibitors singly also revealed dose-dependent decreases in Isg15 expression (S5 Fig). The SphK inhibitors may have off-target effects that could alter Isg15 expression, so we used siRNAs to reduce expression of the sphingosine kinases. SiRNAs against both kinases in combination, or against SphK1 alone, caused a significant reduction in Isg15 expression (Fig 3G and 3H). The data therefore show that deletion of *SphK* genes, use of SphK inhibitors, and use of SphK siRNAs all reduce Isg15 levels in a manner similar to deletion of flotillins.

### Flotillins recruit Sph to membranes

**Fig 4:**
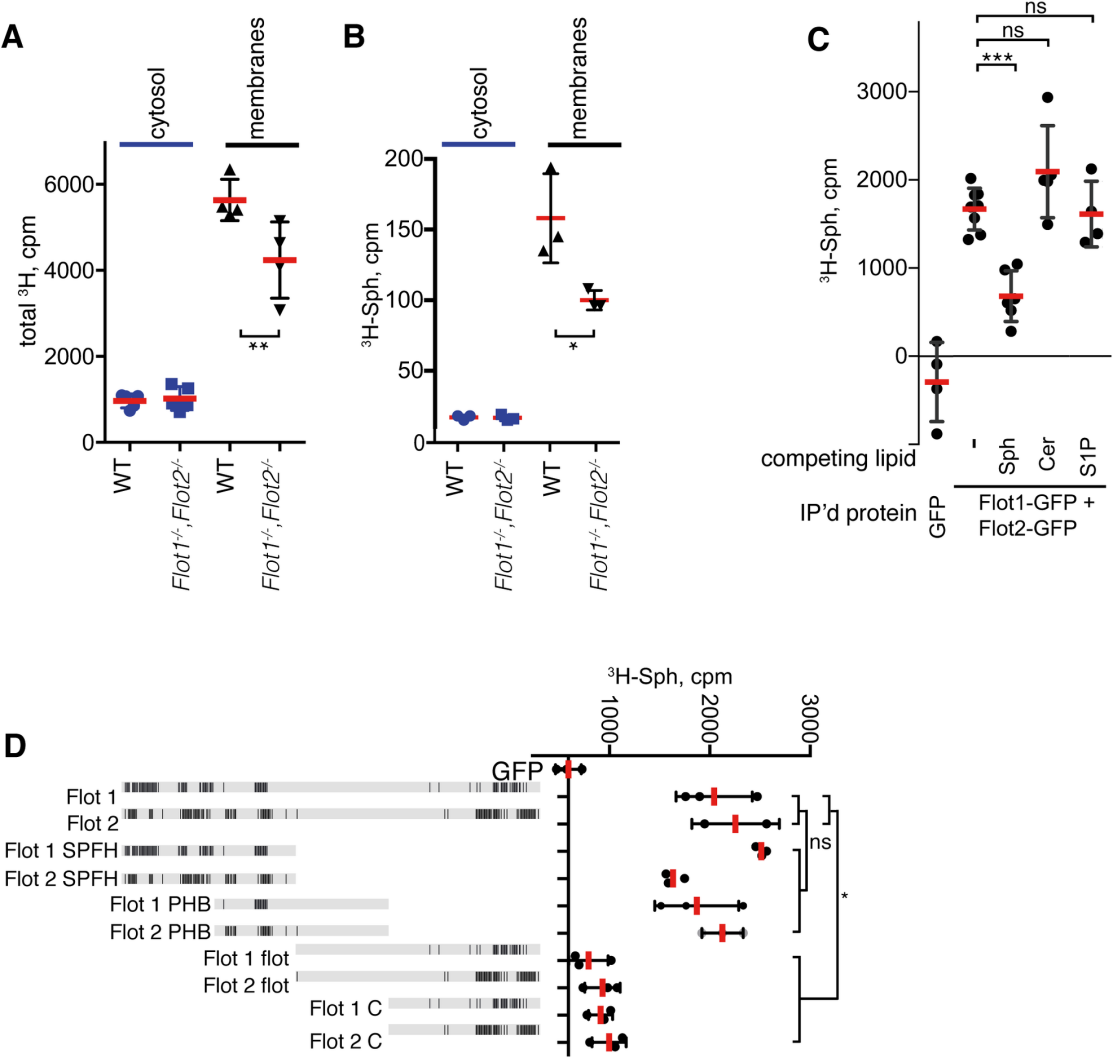
Flotillins bind to ^3^H-Sph. **A.** ^3^H-Sph distribution into cytosolic and membrane franctions in WT and *Flot1*^-/-^, *Flot2*^-/-^ MEFs. Data are means with SD, pooled data from 2 separate experiments, each point is a separate replicate, Student’s t-test. **B**. As A, but lipid extracts from membrane and cytosolic fractions were further fractionated by thin-layer chromatography before isolation of the specific Sph band. **C**. ^3^H-Sph binding to *in vitro* translated and affinity purified flotillin-1-GFP and flotillin-2-GFP proteins. ^3^H-Sph binding was competed with excess of cold Sph, ceramide (Cer) or S1P. Each point is a separate replicate, pooled data from 3 separate experiments. Data are means with SD, Student’s t-test. **D**. ^3^H-Sph binding to *in vitro* translated and affinity purified flotillin-1-GFP and flotillin-2-GFP truncation mutants. The graphical representations of flotillin 1 and flotillin 2 sequences show in black regions of hydrophobicity > 0.25 using Kyte–Doolittle values averaged over a window of 15 amino acid residues. Precise sizes of all constructs are given in the Methods section. Statistical comparisons apply one way ANOVA with Tukey’s multiple comparison test.

Given the reduced S1P levels in flotillin knockout cells and mice, we asked whether flotillins might regulate the distribution of relevant sphingolipids such as Sph We followed the subcellular distribution of Sph in control and knockout cells using a biochemical approach and radiolabelled Sph. Addition of ^3^H-Sph to cells on ice results in rapid accumulation of the lipid in cells. Sub-cellular fractionation assays revealed that ^3^H counts associated with total membranes were reduced in *Flot1*^-/-^, *Flot2*^-/-^ cells (Fig 4A). Separation of lipids from membrane and cytosolic fractions using thin layer chromatography allowed isolation of the specific ^3^H-Sph band. Scintillographic counting confirmed that ^3^H-Sph is specifically reduced in the *Flot1*^-/-^, *Flot2*^-/-^ membrane fractions (Fig 4B).

The experiments presented above show that Sph is recruited to membranes less efficiently in the absence of flotillins. This could be explained by binding of flotillins to Sph. We analysed binding of radiolabelled Sph to *in vitro* translated and affinity purified flotillin1-GFP and flotillin2-GFP. Compared to control protein GFP, both flotillin-GFP proteins had more affinity to Sph (Fig 4C). Excess unlabelled Sph competed with ^3^H-Sph binding to flotillins, whereas other sphingolipids ceramide and S1P did not (Fig 4C). This suggests that association of Sph and flotillin is specific, rather than merely resulting from the hydrophobic properties of both molecules. We used the same *in vitro* binding assay to ascertain which domain of the flotillins binds to Sph (Fig 4D). The N-terminal SPFH domain bound to Sph with a similar efficiency to full-length flotillins, as did an overlapping region with high homology to another SPFH domain protein, prohibitin [29]. Constructs comprising the rest of the protein, or just the hydrophobic region at the C-terminus of both proteins, did not show significant Sph-binding affinity (Fig 4D). These data suggest that the SPFH domain of flotillins is a Sph-binding module. However, these *in vitro* experiments do not yield information on the stoichiometry of the flotillin-sphingosine interaction and may not recapitulate the behaviour of flotillins in intact membranes. Accordingly, we sought additional confirmation that Sph associates with flotillin microdomains *in vivo*.

**Fig 5:**
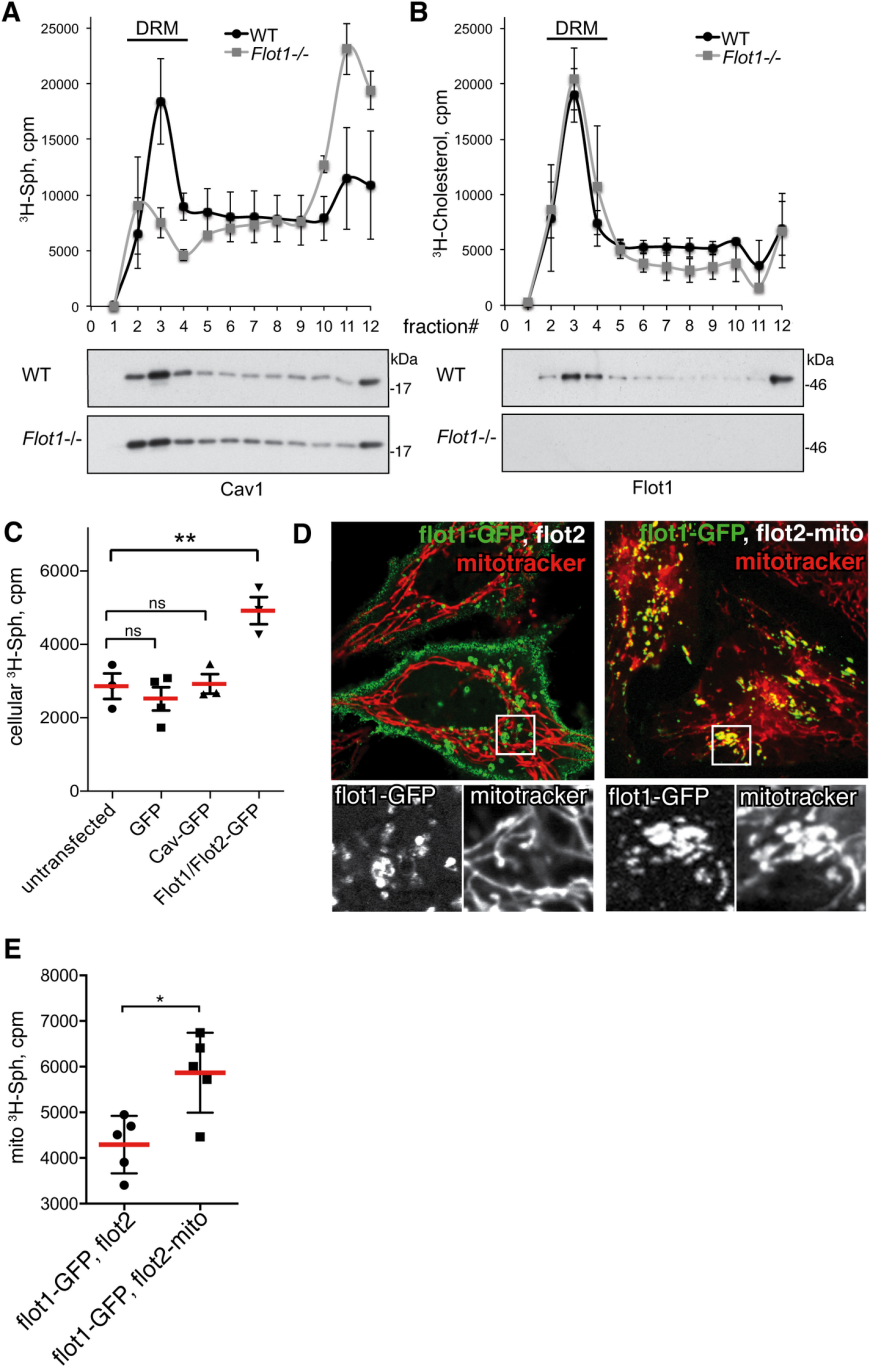
Flotillins recruit ^3^H-Sph to specific cellular membrane domains. **A.** Detergent resistant membrane (DRM) isolation by sucrose gradient centrifugation of ^3^H-Sph labelled WT and *Flot1*^-/-^ primary MEFs. ^3^H-Sph levels in fractions from the gradient were analysed by liquid scintillation counting. Data are means with SEM, N = 3 separate experiments. Western blots below the graph shows caveolin 1 distribution in the gradient fractions. Full scans of all blots are shown in S3 Data File. **B**. ^3^H-cholesterol distribution into DRMs in WT and *Flot1*^-/-^ MEFs. Data are means with SEM. Western blots below the graph show flotillin 1 distribution in the gradient fractions. **C**. Overexpression of flotillins increases Sph accumulation in HeLa cells. HeLa cells were transfected with GFP-tagged proteins and the overexpressing cells were FACS sorted and incubated with ^3^H-Sph. Radioactivity was measured with liquid scintillation. Data are means with SEM, N = 3 separate experiments. One way ANOVA with Dunnett’s multiple comparisons test, Flot-GFP expressing cells compared to all other groups. **D**. Ectopic re-localisation of flotillins to mitochondria. Flotillin-1-GFP is co-expressed with either flotillin 2 or flot2-mito, the latter two constructs lack a fluorescent tag. Mitochondria are visualised using mitotracker dye, the lower panels show magnified views of both fluorescence channels within the boxed areas. **E**. Quantification of ^3^H-Sph incorporation into mitochondria. Cells were loaded with ^3^H-Sph for 5min before isolation of mitochondria, and counting of radioactivity in the isolated mitochondria by liquid scintillation. Bars are means and SD, N = 5, Student’s t-test.

Flotillin microdomains are highly resistant to membrane solubilisation with non-ionic detergents [30], so one would predict that if Sph is associated with these microdomains it should itself display flotillin-dependent recruitment to detergent resistant membrane (DRM) fractions [31, 32]. Analysis of isolated DRMs from MEFs that had been incubated with radiolabelled Sph showed it to co-fractionate with flotillins in DRMs in control cells. Importantly, recruitment of Sph to DRMs was lost in *Flot1*^-/-^ cells (Fig 5A). Another DRM lipid, cholesterol [32], and the DRM protein caveolin [33], distributed into DRMs in both WT and *Flot1*^-/-^ MEFs (Fig 5A and 5B), demonstrating that the general physico-chemical properties of DRMs were not affected in flotillin knockout cells. Flotillin-dependent recruitment of Sph to DRMs can explained by direct binding of flotillins to Sph, but it is also possible that flotillins alter the local physical properties of the bilayer so as to promote Sph recruitment. In either eventuality, the conclusion is that flotillins act to promote local membrane recruitment of Sph.

As an further test of whether flotillins associate with Sph we overexpressed flotillin-GFPs in HeLa cells, and asked whether this gain-of-function is sufficient to increase recruitment of Sph. Overexpression of flotillins resulted in increased cellular Sph accumulation, but overexpression of caveolin-1-GFP, which has similar membrane topology and multimerisation properties to flotillins, does not (Fig 5C). This experiment did not require fractionation or any other biochemical perturbation, and so provides additional evidence that flotillin microdomains can indeed recruit Sph within cells.

We tested the possibility that flotillin microdomains recruit Sph using ectopic re-localisation of the flotillins. Either flotillin1-GFP plus flotillin2, or flotillin1-GFP plus a mitochondrially targeted version of flotillin2 (flot2-mito, [34]) were transiently expressed in HeLa cells. In the former case flotillin1-GFP was, as predicted, localised to the plasma membrane and intracellular organelles that we know to be late endosomes / lysosomes [13], whilst co-expression with flot2-mito resulted in the flotillin1-GFP being efficiently redistributed to mitochondria (Fig 5D). The fact that flot2-mito causes this redistribution of normal flotillin1-GFP demonstrates that complexes containing the two proteins can assemble on mitochondrial membranes. Transiently transfected cells were loaded with ^3^H-Sph for 5min, and then mitochondria were purified. Even though transfection efficiency in these experiments was in the range 40–50%, there was a clear and significant increase in mitochondrial ^3^H-Sph in the cells expressing mitochondrially-targeted flotillins (Fig 5E). This provides direct evidence that sub-cellular redistribution of flotillin microdomains causes consequent redistribution of ^3^H-Sph.

## Discussion

The data presented here lead to three main observations. First, there is less S1P in flotillin knockout cells and tissues. Second, cellular phenotypes of flotillin knockouts can be recapitulated by genetic and pharmacological perturbation of sphingosine kinase activity, and conversely some phenotypes previously attributed to loss of S1P activity are also observed in flotillin knockouts. Third, sphingosine is recruited to membranes in the presence of flotillins, and gain of flotillin function leads to enhanced Sph recruitment.

Our data imply that one function of flotillins could be to control the membrane localisation of Sph. It will be important to investigate further whether flotillin-dependent recruitment of Sph impacts on S1P production directly, as at present we do not exclude the possibility that further factors are involved. Similarly, although we show a correlation between phenotypes of flotillin knockouts and those caused by perturbing SphK activity, further experiments are needed to determine more precise underlying mechanisms.

We did not observe changes in S1P levels in RBC pellets or serum from flotillin knockout mice. The differential effects on S1P levels in tissues and in blood that we observed imply that mechanisms mediating S1P production are different in these contexts. We speculate that this may reflect the lack of sub-cellular organelles, and hence lack of sub-cellular compartmentalisation, in erythrocytes. S1P levels in blood, according to both our own and previous measurements, are higher than in the surrounding tissue, and this S1P gradient is important for many of the functions of S1P [6, 22, 35]). It is therefore difficult to interpret the absolute amount of S1P in flotillin knockout tissues observed by mass spectrometry, as we did not remove blood from the vasculature. It is clear, however, that in multiple cell types and tissues less S1P is detected in flotillin knockouts than in control samples.

We note that interpretation of the significance of changes in observed levels of Sph, S1P, or other sphingolipid metabolites, is hampered by the dynamic inter-conversion of lipid species. Future experiments quantifying flux through sphingolipid metabolic pathways in different cell types may provide further insight.

Both altered Isg15 levels and specific changes in histone acetylation are observed in flotillin knockout cells, cells treated with SphK inhibitors, and in SphK knockouts. Similar changes in histone acetylation were observed in cells treated with SphK siRNAs [24], and we report that SphK siRNAs also reduce Isg15 expression levels. S1P acts as an extracellular signal via 5 G-protein coupled receptors, and may also bind to intracellular targets [2, 6], and it will be important to ascertain the mechanism by which the signalling activity of S1P explains the phenotypes we report. The differential roles of SphK1 and SphK2 will also require further investigation, as it is possible that the inhibitors we have used have different efficacies, and siRNA treatment produced more efficient knockdown of SphK1 than SphK2.

Co-regulation of the transcription of interferon-sensitive genes such as ISG15, and of histone acetylation is consistent with a body of literature implicating histone acetylation status in interferon responsiveness [36]. The situation is complicated by the fact that in some situations, for example viral infection, loss of histone acetylation is linked to increased, rather than decreased, ISG15 levels [37]. It may be that these effects are complex and context dependent [36]. Intriguingly, previous observations in embryonic trophoblast cells that may in this respect function analogously to our MEF cultures show that in this case decreased histone acetylation is indeed causally linked to repression of interferon-sensitive genes [38].

Our new data suggesting a link between flotillins and S1P production fit well with several previous observations. Previously we have shown that neutrophil migration in vivo is impaired in flotillin knockout mice, and given the role of S1P in inflammation and leukocyte migration it is likely that reduced S1P levels contribute to this phenotype [3, 39]. There is a large body of literature implicating flotillins in endocytosis [16, 40]. Intriguing observations associate S1P levels and recruitment of SphK1 with formation of endocytic structures at the plasma membrane. S1P may therefore regulate rates of endocytosis [41–43].

Flotillins interact with the cytosolic leaflet of the plasma membrane via a conserved SPFH domain, and we find that this domain has Sph-binding affinity [44, 45]. Given the technical difficulties in demonstrating direct association between specific lipids and proteins in intact cell membranes, it may be that in cells the SPFH domain interacts with a wider range of lipids than we have been able to access in our experiments. There is good evidence that mitochondrial prohibitin 2, which has the same domain, binds to S1P to regulate mitochondrial metabolism [5]. That study therefore provides prior evidence that the SPFH domain may have Sph or S1P-binding activity. Finally, there is evidence for organisation of sphingolipids in specific microdomains within the plasma membrane, and it will be important to ascertain whether flotillins play any role in this more general sphingolipid organisation [46].

## Methods

### Animal experiments

All mouse experiments were carried out under a personal project licence granted by the UK Home Office, and were authorised by the MRC-LMB Ethical review committee. Sample sizes were chosen using estimates of variance from previous experimental data. No animals were excluded from analysis in any of the experimental cohorts. Cohorts were not formally randomised but were selected to contain individuals from multiple litters. Lipidomic analysis was carried out blind to animal genotype. All methods were performed in accordance with the relevant guidelines and regulations.

### Reagents and antibodies

Sphingosine, ceramide, and sphingosine-1-phosphate were from Avanti Polar lipids, SphK1 inhibitor PF-543 from Pfizer, SphK2 inhibitor ABC294640 was from Active Biochemicals. MitoTracker Deep Red was from Life Technologies Ltd. Antibodies recognising the following proteins were used: flotillin 1 (610820), flotillin 2 (610384) from BD Transduction Laboratories, SphK1 (12071) and Isg15 (2743) from Cell Signaling, Histone-H3 K9ac (ab10812), Histone H3 K14ac (ab52946), Histone H4 K5ac (ab51997), Histone H3 (ab10799), Histone H4 (ab10158) all from Abcam, SphK2 (17096) from Proteintech Group. All experiments using antibodies for Western blotting were performed at least twice and representative blots are shown. Quantification was carried out by scanning and densitometry using Image J.

### Cell culture and treatments

HeLa cells were obtained from ATCC and were tested for mycoplasma contamination. Primary MEFs were tested for mycoplasma contamination and were grown in DMEM-GlutaMAX (Gibco) with heat inactivated 10% foetal bovine serum (FBS, HyClone, Thermo Scientific) and penicillin/streptomycin. SphK inhibitors were used in concentrations of 10 μM (PF543) and 50 μM (ABC294640). The cells were washed with PBS before cell lysis in Western blotting sample buffer.

### Lipid mass spectrometry

For glycerophospholipid analysis, 1 million of MEF cell pellets were suspended in 1.5ml methanol. Lipid standard mixture in 1.5ml of LCMS-grade water and 3ml chloroform were added in. The mixture was subjected to Folch extraction. After collection of the lower phase, the upper phase was re-extracted with 3 ml synthetic lower phase. The lower phase from both extractions was combined and dried under vacuum and re-dissolved in 60 μl chloroform. 7 μl was injected for LC/MS/MS analysis. A Thermo Orbitrap Elite system (Thermo Fisher) hyphenated with a five-channel online degasser, four-pump, column oven, and autosampler with cooler Shimadzu Prominence HPLC system (Shimadzu) was used for lipids analysis. In detail, lipid classes were separated on a normal phase silica gel column with hexane/dichloromethane/chloroform/methanol/acetanitrile/water/ethylamine solvent gradient based on the polarity of head group. High resolution (240k at m/z 400) / accurate mass (with mass accuracy <5ppm) and tandem MS (CID fragmentation) were used for molecular species identification and quantification. The identity of lipid was further confirmed by reference to appropriate lipids standards. Lipid standards used, and the amount of each standard analysed, are given in the relevant Supplementary Data files.

For sphingolipid extraction, tissue samples were snap-frozen in liquid nitrogen immediately after dissection from freshly culled mice. Red blood cell pellets were obtained by cardiac puncture, collection in EDTA tubes and centrifugation before immediate snap freezing. 2 million MEF cells, 30 mg of pulverised mouse brain tissue, 50 mg of liver tissue, adipose tissue, or muscle tissue fine particles in liquid nitrogen, or 20 μl mouse serum, or red blood cell pellet were suspended in 1 ml LCMS grade water, 2 ml of n-butanol, and 100 ng C17-S1P, 200 ng C17-SM, 100 ng C17-Cer, and 50 ng C17-Sph were added in. The mixed samples were vortexed for 15 seconds and sonicated in water bath for 2 minutes. After spin at 3750 rpm 4°C for 5 minutes, the upper organic phase was collected. The lower aqueous phase was re-extracted with 1 ml of n-butanol. The combined upper organic phases were dried under vacuum and re-dissolved in 60 μl chloroform/methanol/water 2:5:1 (v/v/v). 7 μl of the lipid extract was injected onto a Gemini–NX- 3u C18 2.0x150mm column (Phenomenex) for LC-MS/MS analysis with Shimadzu Prominence HPLC system hyphenated with AB Sciex 4000 QTRAP mass spectrometer. Lipid separation was performed with a binary gradient elution with acetonitrile/formic acid/ammonium formate.

### SiRNA treatment

5 million HeLa cells were transfected with 100 nM of siRNA targeting human flotillin 1 (J-010636-05) and flotillin 2 (J-003666-09), SPHK1 (J-004172-10) and SPHK2 (J-004831-10), or 200 nM non-targeting siRNA, (all from Dharmacon) using oligofectamine (Invitrogen). After 72 hours the cells were washed and scraped in PBS, spun down, and frozen until analysis by lipid mass spec as above.

### Isolation of nuclei

WT and flotillin knockout MEFs were suspended in 50 mM Tris, pH 7.4, 250 mM sucrose, 10 mM KCl, and 1 mM DTT and broken with repeated pulls through 23G needle. Unbroken cells were removed by a 100 x *g* spin for 10 min, after which nuclei were separated by 500 x *g* spin for 10 min. Purified nuclei were suspended in 100 mM Tris, 250 mM KCl, and 10 mM MgCl_2_, and incubated with 20 μM of either sph or S1P at 37°C for 5 or 20 min. Histone acetylation was analysed by western blotting.

### Membrane and DRM analysis

Primary MEFs were labelled with 0.2 μCi/ml of 3-^3^H-D-erythro-sphingosine for 10 min on ice. For analysis of Sph levels in cell cytosol and membranes, the cells were suspended in 10 mM Hepes and broken with repeated pulls through 23G needle. Unbroken cells and nuclei were removed by a 500 x *g* spin for 10 min, after which cell membranes were separated from cytosol with a 150 000 x *g* spin of post-nuclear supernatant for 30 min. Radioactivity in the cytosol and membranes dissolved in 1% SDS was counted with Liquid Scintillation Analyzer (Perkin Elmer). For DRM isolation, after ^3^H-Sph or 1 μCi/ml 1,2-^3^H-cholesterol (Perkin Elmer) labelling for 10 minutes, the cells were washed, and solubilized in 1% Triton X-100, 50 mM Tris pH 7.4, 150 mM NaCl on ice for 30 min. 1 ml of lysate was mixed with 1 ml 85% sucrose, and this suspension was overlayed with 35% sucrose and 5% sucrose. After 180 000 x *g* spin with SW40 Ti rotor for 16 h, the 35%-5% interface (DRM) and other fractions were collected and radioactivity was counted as above.

### Thin-layer chromatography

Lipids were extracted with the Bligh and Dyer method and separated on silica gel TLC plates (Merck) with Chloroform/Methanol/Water (65:25:4). Sph standard was visualised using ninhydrin solution (Sigma), and corresponding Sph spots in samples were scraped off into vials for scintillation counting (Perkin Elmer).

### Sph-binding assay

PCR products with T7 primer and kozac sequences of GFP, flotillin1-GFP, flotillin2-GFP, and different domains of flotillins with C-terminal GFP-tags were used for *in vitro* transcription/translation (T7 TNT Quick Coupled Transcription/Translation Systems, Promega). The different truncation mutants shown in Fig 4 correspond to residues 3–185 in Flot 1 SPFH, 186–427 Flot 1 flot, 84–266 Flot 1 PHB, 267–467 Flot1 C, 5–188 in Flot 2 SPFH, 189–427 Flot 2 flot, 87–268 Flot 2 PHB, 269–467 Flot 2 C. Protein mixture was diluted with 3% bovine serum albumin (BSA) in PBS, and 0.5 μCi/ml ^3^H-Sph and magnetic anti-GFP microbeads (Miltenyi Biotec) were added for 1 h incubation on ice. The beads were bound on magnetic columns, washed with BSA/PBS and eluted by removing the magnet. Bound ^3^H-Sph was analysed by Liquid Scintillation Analyzer (Perkin Elmer). For lipid competition assay, 10 μM of Sph, S1P, or Cer was added to the translated proteins 5 min before adding ^3^H-Sph and anti-GFP microbeads.

### Mitochondrially targeted flotillins

Flotillin 2 was cloned into AgeI and EcoRI sites of pU39 with a C-terminal transmembrane domain of monoamine oxidase (MOA), which is sufficient for targeting to the outer mitochondrial membrane [34, 47]. HeLa cells were transfected with flotillin 2-MOA (‘flot2-mito’) and flotillin-1-GFP. Mitochondrial targeting was determined by confocal microscopy using 20 nM MitoTracker Deep Red (Molecular probes). To elucidate sphingosine recruitment on mitochondria, 10^7^ Hela cells were transiently transfected, the cells were harvested and chilled on ice, and incubated with 1 μCi/ml ^3^H-Sph for 5 min. After washes with cold PBS, mitochondria were purified using Mitochondria isolation kit (Thermo Scientific) and ^3^H-Sph was quantified with liquid scintillation analyser (Perkin Elmer).

### Measurement of cellular Sph accumulation

To analyse overexpression effect on cellular ^3^H-Sph accumulation, HeLa cells were transfected with GFP-tagged proteins using Fugene (Promega), and after 18 h the cells were trypsinised and fluorescent cells were sorted by flow cytometer. 10^5^ cells were incubated with 0.4 μCi/ml ^3^H-Sph in medium for 10 min on ice, washed with ice cold PBS, lysed in 1% SDS, and the radioactivity was measured with Liquid Scintillation Analyzer (Perkin Elmer).

### SILAC

Primary MEFs were grown in DMEM lacking Lysine, Arginine and Leucine (Sigma) with 10% dialyzed FBS, and normal isotopic Lys, Arg, Leu (Sigma) for the light sample, or Leu, ^13^C_6_^15^N_2_ Lys, ^13^C_6_^15^N_4_ Arg (Sigma) for the heavy sample. Cells were grown until > 99% incorporation of isotopic amino acids, which was confirmed by LC-MS/MS. Cells were trypsinised and pooled together in 0.25 M sucrose, 1 mM EDTA, 20 mM Hepes buffer before being broken by pulling through a 21G needle. The plasma membrane fraction was isolated by flotation through an iodixanol (Axis-Shield) discontinuous density gradient and the samples were collected from the 17.5%-25% interphase. Proteins were methanol precipitated and samples were prepared for mass spectrometric analysis using a filter-aided sample preparation method, essentially as described previously [48], using Lys C and Trypsin digest. The resulting peptides were fractionated by liquid isoelectric focusing using Agilent 3100 OFFGEL fractionator (Agilent Technologies), and analysed by LC-MS/MS and quadrupole ion trap mass spectrometer (Orbitrap LTQ XL, ThermoScientific). Data analysis was performed using MaxQuant software [49], which utilizes Mascot search engine programme (Matrix Science) for peptide identification.

### Statistical tests

Comparison of paired data sets was carried out using Student’s t-test. Where one data set was to be compared with multiple sets comparison was carried out using one-way ANOVA with Dunnett’s multiple comparisons test. The nature of the test used in specific cases is given in the relevant Fig legend. In all cases significance was assessed as p<0.05, and expressed p<0.05 = * p<0.01 = **, p<0.001 = ***, p<0.0001 = ****.

## Supporting information

S1 Data File Quantitative lipid mass spectrometry using calibration with defined standards, of WT and flotillin knockout MEFs. Sphingolipid species were analysed.
(XLSX)

S2 Data File Quantitative lipid mass spectrometry using calibration with defined standards, of WT and flotillin knockout MEFs. Lipid species analysed are detailed within the spreadsheet.
(XLSX)

S3 Data File Full scans of Western blots. (PDF)

S1 Fig Amounts of lipids isolated from flotillin knockout cells. Quantitative lipid mass spectrometry using calibration with defined standards, of WT and flotillin knockout MEFs. 1x106 cells were analysed in each sample. PA, phosphatidic acid; PC, phosphatidylcholine; PE, phosphatidylethanolamine; PG, phosphatidylglycerol; PI, phosphatidylinositol; PS, phosphatidylserine, CH, cholesterol; DAG, diacylglycerol; FFA, free fatty acids; LPC, lysophosphatidic acid. Data from 2 or 4 biological replicates, each data point being derived from a separate experiment. Raw data are in S2 Data File. Bars are mean.
(DOCX)

S2 Fig Sphingolipid species are not altered by deletion of flotillin genes. Quantitative lipid mass spectrometry using calibration with defined standards, of WT and flotillin knockout MEFs. 1x10^6^ cells were analysed in each sample. Cer = ceramide, SM = sphingomyelin. The data shown are means from two replicates in a single analysis, the analysis was repeated twice with the same result. Raw data for these and further lipid species are given in S1 Data File.
(DOCX)

S3 Fig Sphingosine levels in tissues from flotillin knockout mice. Sph levels in mouse tissues as shown, determined by quantitative lipid mass spectrometry. Bars are means and SD, each point represents a single sample from a different animal.
(DOCX)

S4 Fig Changes in mRNA levels in *Flot1-/-* cells. Reduced mRNA levels in Flot1-/- MEFs. Quantitative PCR from cDNA was used to measure amounts of Isg15 and Bst2 mRNAs. The data were normalised to GAPDH expression and expressed as the ratio between control and knockout cells. N = 4, bars SD.
(DOCX)

S5 Fig Sphingosine kinase inhibitors reduce expression of Isg15. Isg15 expression levels in WT MEFs cells treated with SphK1 inhibitor PF543 and with SphK2 inhibitor ABC294640 for 24h as shown.
(DOCX)
