# Supplementary material for: Flotillin proteins recruit sphingosine to membranes and maintain cellular sphingosine-1-phosphate levels
Source: PLoS One. 2018 May 22;13(5):e0197401. doi: 10.1371/journal.pone.0197401 (PMC5963794; doi:10.1371/journal.pone.0197401)

S8 Data File - full scans of all blots

The region shown in the Figure is denoted by a red box

Figure 1C, blot anti-flotillin 1

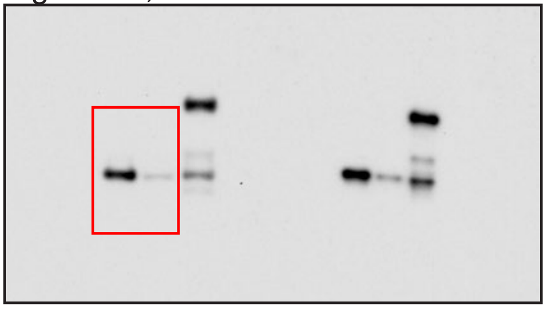

Figure 2A, blot anti-histone H3 K9 actylated

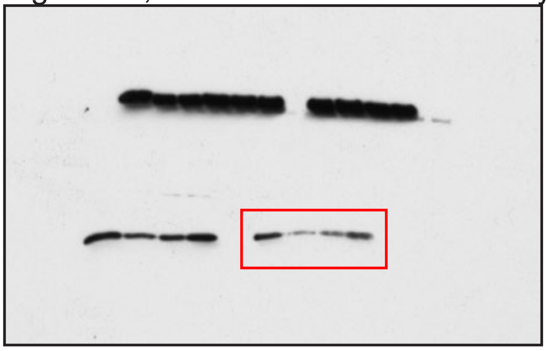

Figure 2A, blot anti-histone H3

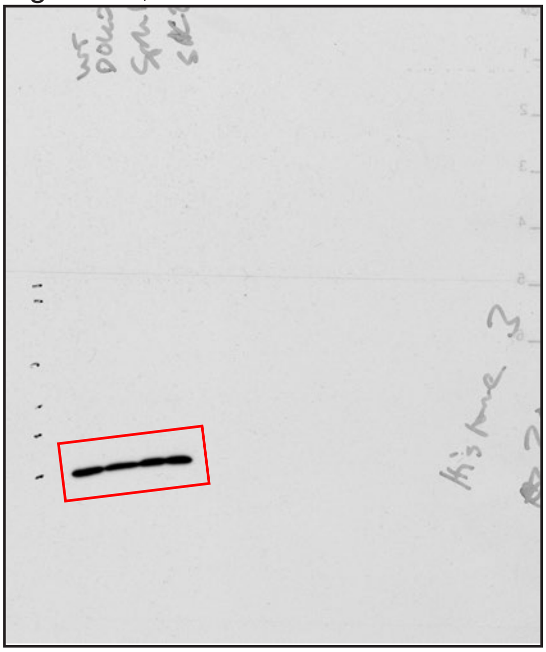

Figure 2C, blot anti-histone H3 K9 actylated

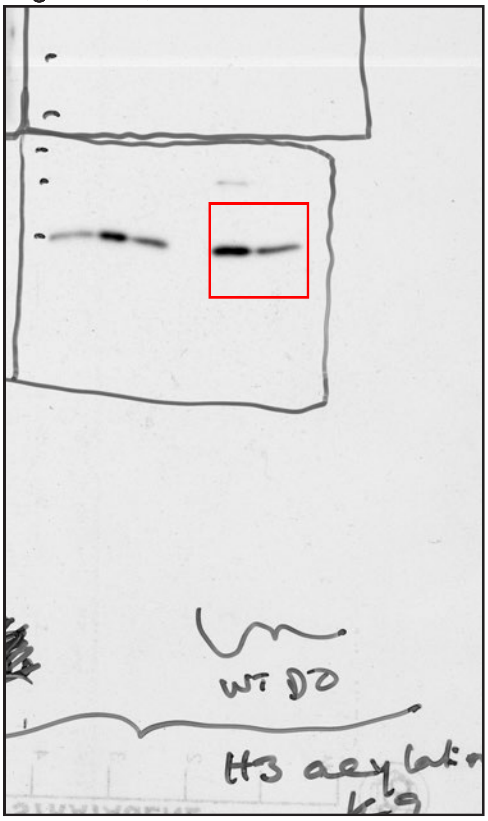

Figure 2C, blot anti-histone H3 K14 actylated

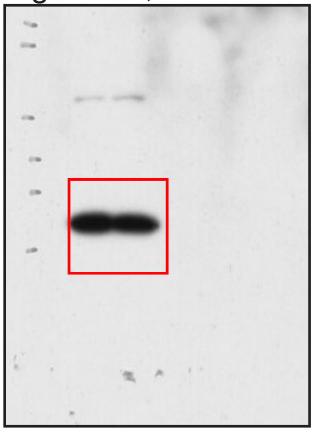

Figure 2C, blot anti-histone H3

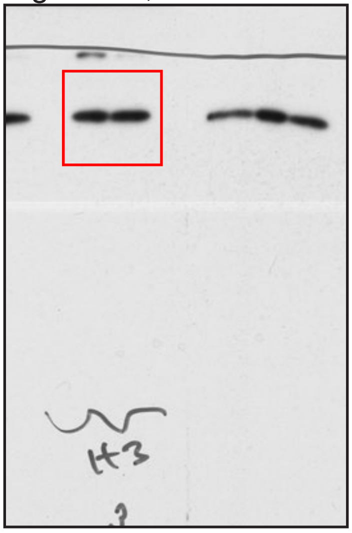

Figure 2C, blot anti-histone H4

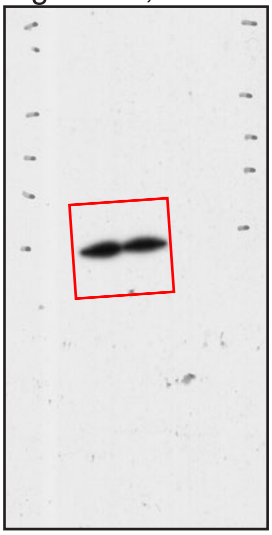

Figure 2E, blot anti-histone H3 K9 actylated

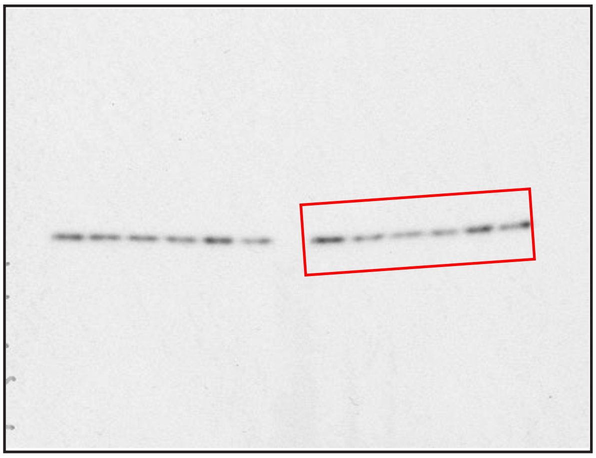

Figure 2E, blot anti-histone H3

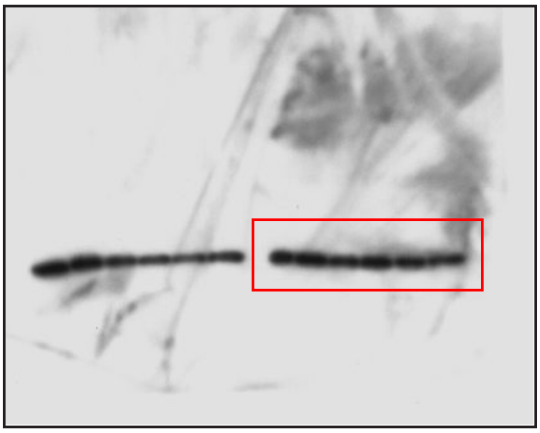

Figure 2G, blot anti-ISG15

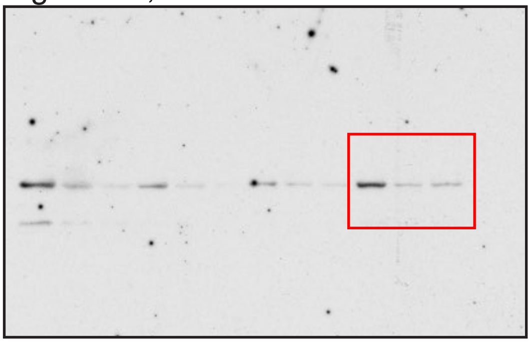

Figure 2G, blot anti-actin

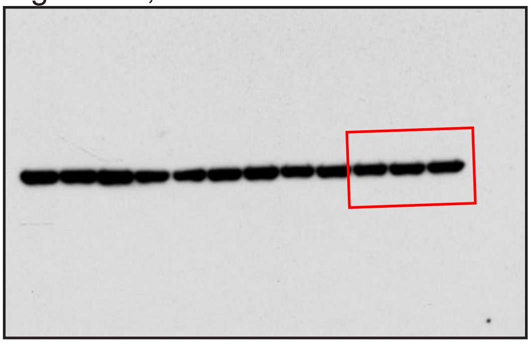

Figure 3A, blot anti-ISG15

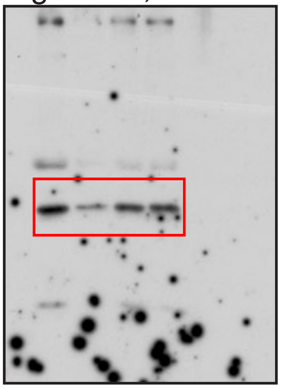

Figure 3A, blot anti-actin

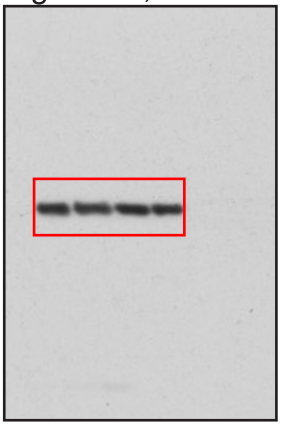

Figure 3B, blot anti-ISG15

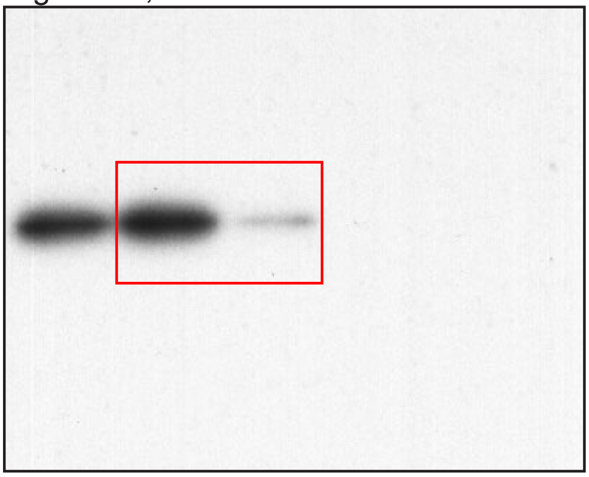

Figure 3B, blot anti-actin

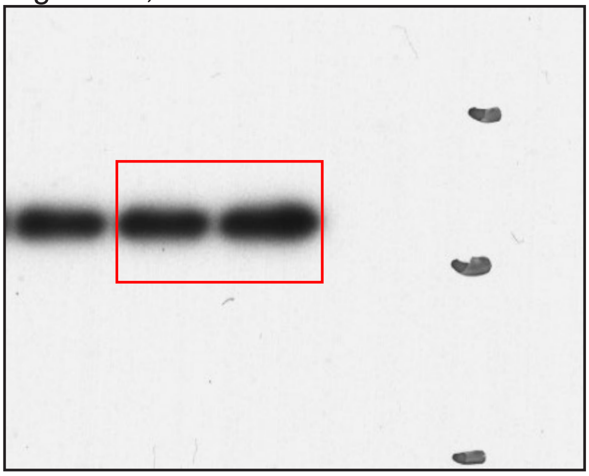

Figure 5A, blot anti-caveolin 1

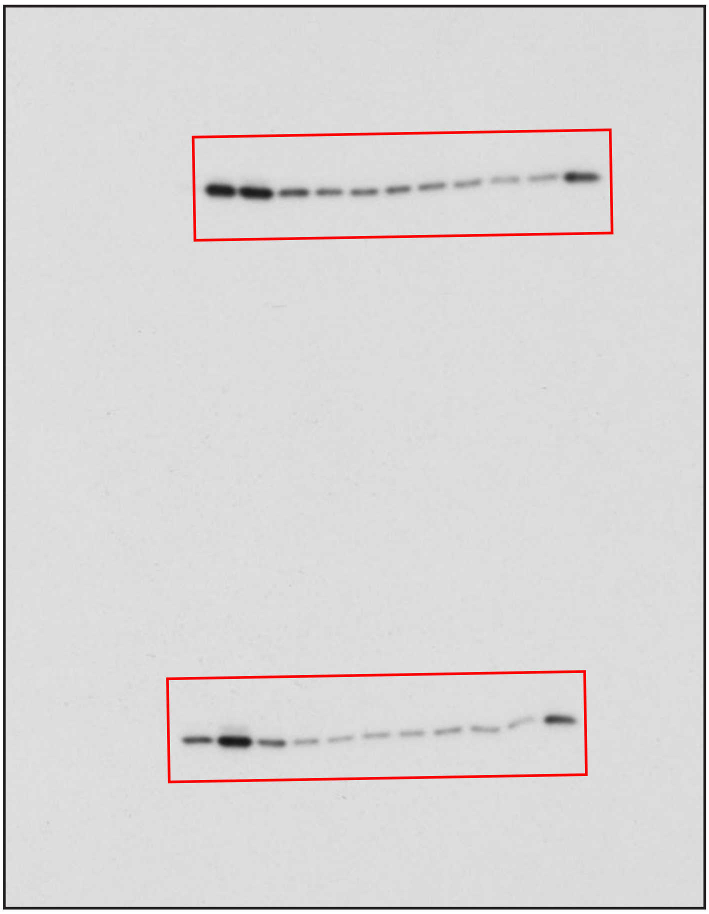

Figure 5B, blot anti-flotillin 1

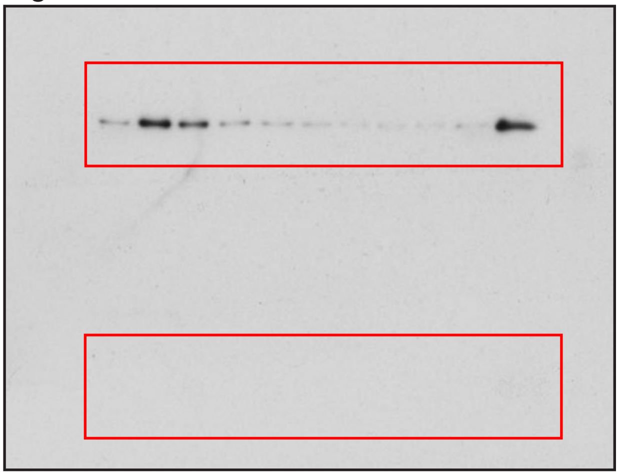

Supplement: S3 Data File — (PDF) [file pone.0197401.s003.pdf]
