# Supplementary material for: Flotillin proteins recruit sphingosine to membranes and maintain cellular sphingosine-1-phosphate levels
Source: PLoS One. 2018 May 22;13(5):e0197401. doi: 10.1371/journal.pone.0197401 (PMC5963794; doi:10.1371/journal.pone.0197401)
Supplement: S1 Fig — Quantitative lipid mass spectrometry using calibration with defined standards, of WT and flotillin knockout MEFs. 1x10^6^ cells were analysed in each sample. PA, phosphatidic acid; PC, phosphatidylcholine; PE, phosphatidylethanolamine; PG, phosphatidylglycerol; PI, phosphatidylinositol; PS, phosphatidylserine, CH, cholesterol; DAG, diacylglycerol; FFA, free fatty acids; LPC, lysophosphatidic acid. Data from 2 or 4 biological replicates, each data point being derived from a separate experiment. Raw data are in S2 Data File. Bars are mean. (DOCX) [file pone.0197401.s004.docx]

**S1 Fig. Amounts of lipids isolated from flotillin knockout cells.** Quantitative lipid mass spectrometry using calibration with defined standards, of WT and flotillin knockout MEFs. 1x106 cells were analysed in each sample. PA, phosphatidic acid; PC, phosphatidylcholine; PE, phosphatidylethanolamine; PG, phosphatidylglycerol; PI, phosphatidylinositol; PS, phosphatidylserine, CH, cholesterol; DAG, diacylglycerol; FFA, free fatty acids; LPC, lysophosphatidic acid. Data from 2 or 4 biological replicates, each data point being derived from a separate experiment. Raw data are in S7 Data File. Bars are mean.
