# Supplementary material for: Flotillin proteins recruit sphingosine to membranes and maintain cellular sphingosine-1-phosphate levels
Source: PLoS One. 2018 May 22;13(5):e0197401. doi: 10.1371/journal.pone.0197401 (PMC5963794; doi:10.1371/journal.pone.0197401)
Supplement: S2 Fig — Quantitative lipid mass spectrometry using calibration with defined standards, of WT and flotillin knockout MEFs. 1x10^6^ cells were analysed in each sample. Cer = ceramide, SM = sphingomyelin. The data shown are means from two replicates in a single analysis, the analysis was repeated twice with the same result. Raw data for these and further lipid species are given in S1 Data File. (DOCX) [file pone.0197401.s005.docx]

**S2 Fig. Sphingolipid species are not altered by deletion of flotillin genes.** Quantitative lipid mass spectrometry using calibration with defined standards, of WT and flotillin knockout MEFs. 1x10^6^ cells were analysed in each sample. Cer = ceramide, SM = sphingomyelin. The data shown are means from two replicates in a single analysis, the analysis was repeated twice with the same result. Raw data for these and further lipid species are given in S6 Data File.
