# Supplementary material for: Flotillin proteins recruit sphingosine to membranes and maintain cellular sphingosine-1-phosphate levels
Source: PLoS One. 2018 May 22;13(5):e0197401. doi: 10.1371/journal.pone.0197401 (PMC5963794; doi:10.1371/journal.pone.0197401)
Supplement: S3 Fig — Sph levels in mouse tissues as shown, determined by quantitative lipid mass spectrometry. Bars are means and SD, each point represents a single sample from a different animal. (DOCX) [file pone.0197401.s006.docx]

**S3 Fig. Sphingosine levels in tissues from flotillin knockout mice.** Sph levels in mouse tissues as shown, determined by quantitative lipid mass spectrometry. Bars are means and SD, each point represents a single sample from a different animal.
