# Supplementary material for: Flotillin proteins recruit sphingosine to membranes and maintain cellular sphingosine-1-phosphate levels
Source: PLoS One. 2018 May 22;13(5):e0197401. doi: 10.1371/journal.pone.0197401 (PMC5963794; doi:10.1371/journal.pone.0197401)
Supplement: S4 Fig — Reduced mRNA levels in Flot1-/- MEFs. Quantitative PCR from cDNA was used to measure amounts of Isg15 and Bst2 mRNAs. The data were normalised to GAPDH expression and expressed as the ratio between control and knockout cells. N = 4, bars SD. (DOCX) [file pone.0197401.s007.docx]

**S4 Fig. Changes in mRNA levels in *Flot1-/-* cells**. Reduced mRNA levels in Flot1-/- MEFs. Quantitative PCR from cDNA was used to measure amounts of Isg15 and Bst2 mRNAs. The data were normalised to GAPDH expression and expressed as the ratio between control and knockout cells. N=4, bars SD.
