# Supplementary material for: Flotillin proteins recruit sphingosine to membranes and maintain cellular sphingosine-1-phosphate levels
Source: PLoS One. 2018 May 22;13(5):e0197401. doi: 10.1371/journal.pone.0197401 (PMC5963794; doi:10.1371/journal.pone.0197401)
Supplement: S5 Fig — Isg15 expression levels in WT MEFs cells treated with SphK1 inhibitor PF543 and with SphK2 inhibitor ABC294640 for 24h as shown. (DOCX) [file pone.0197401.s008.docx]

**S5 Fig. Sphingosine kinase inhibitors reduce expression of Isg15.** Isg15 expression levels in WT MEFs cells treated with SphK1 inhibitor PF543 and with SphK2 inhibitor ABC294640 for 24h as shown.
